# Our New York Letter

**Published:** 1890-06

**Authors:** 


### Correspondence.

### OUR NEW YORK LETTER.

New York, May 17, 1890.

The old adage, *tempora mutantur et nos mutamus in illos*, is in no other department of knowledge so forcibly or better illustrated than in the science and art of medicine. To keep abreast of the progress that is being made in any one branch of the profession is practically a difficult task, and the practicing physician and surgeon who fails to do so is guilty of almost criminal negligence. As an instance of this progress in the knowledge of abdominal diseases alone, may be cited the tremendous strides that have been recently made in this direction. Since the days of Dr. Alonzo Clark, of New York, who was the first to introduce and advocate it, opium has been regarded as the classical treatment for inflammation of the peritoneum. Based on a wrong conception of the disease, as this mode of treatment is known to have been, the pendulum has now swung to the opposite extreme, and the opium treatment has been supplanted by one in which saline cathartics play the main part. An excellent pathological reason exists for discarding opium to a great extent, in the treatment of peritonitis. The fault of the opium treatment lies in the fact that it prevents intestinal movement and invites consequent adhesions of those organs, while the use of saline cathartics is based upon the principle that they excite peristalsis, and in that way prevent adhesions from taking place. Furthermore, by their derivative action they unload the capillaries of the parts, and tend to lessen the inflammation. Opium also masks the progress of the malady until it becomes too late to resort to operative procedures, if deemed advisable for the cure of the disease.

Under the saline treatment noticeable improvement is to be expected within the first 48 hours, this being indicated by a subsidence of the tympanites and swelling of the abdomen.

After the performance of laparotomies, numerous instances have occurred where the pedicle of the tumor that had been removed, formed adhesions with the intestine after the closure of the wound. The pain due to these adhesions was so intense as to necessitate a second, and oftentimes a third surgical procedure for the relief of this one symptom. One eminent gynecologist of this city, to overcome this difficulty, advocates the use of a saline cathartic a few hours after the operation, while another, and no less eminent one, recommends its use just before operative procedures have been commenced.

The saline most in favor here is the sulphate of magnesia, which must be given in sufficiently large doses to produce a copious watery discharge from the intestine. As a result of this, the inflammatory products already thrown out are eliminated through the natural channels, the pain is relieved, and, owing to the peristaltic action of the bowels, the occurrence of adhesions is prevented. Judged from this physiological standpoint, it can be readily understood why this method of treating peritonitis should have so many warm advocates among the gynecologists of New York.

It is, of course, to be understood that the saline mode of treatment herein referred to applies to all forms of peritonitis, and not exclusively to that dependent upon abdominal operation alone. It must also be borne in mind that there are some forms of this disease where the surgeon's knife alone will be of any avail in effecting a cure.

Dr. Bull operated recently at the New York Hospital, on a patient who presented a most interesting and instructive pathological lesion. He was a colored man 70 years of age, and at the time of the operation had an emaciated and cachectic appearance. About six years ago he noticed for the first time a slight swelling on the left side of the chest. This was accompanied with no pain or local irritation of any sort, and remained quiescent for a period of some four years. At the end of that time, it began to grow very rapidly, without involving the deeper tissues of the skin or underlying fat. Dr. Bull stated that the tumor undoubtedly started as a papilloma, but when the man reached that period of life when the skin was predisposed to carcinomatous growths, the original benign tumor took on a malignant character. An operation was undertaken in this case, not from any hope of a possible cure being brought about by extirpation of the neoplasm, but to relieve the patient of the excessive pain he was suffering, and to arrest the profuse and exhausting hemorrhages.

Prof. Fluhrer, of the N. Y. Polyclinic, recommends the use of the following injection in the treatment of acute gonorrhœa:

℞ Potass. Permang. gr. one-sixth.
Zinc. Sulph. gr. one-eighth.
Aquae distil. ℥ j

This should be varied in strength to suit each case, and sometimes it may be desirable to omit the zinc, or increase the strength of the permanganate to a violet hue, but it should never be made so strong as to cauterize the mucous membrane of the urethra. The injection should not smart the parts for more than a few minutes. Under its use, he says, the discharge is quite promptly controlled. It should at first be repeated three or four times daily, and as the patient improves, it should be used less frequently; but the injection should be continued once a night at least, for one week after the subsidence of all symptoms. This same injection has a wide range of usefulness in the different stages of the disease. It may be employed in inflammation of the deeper urethra by means of a catheter inserted down to the compressor urethræ. In the very acute stages of gonorrhœa, to allay the great irritability that is present, he recommends instillations of a 4 per cent. solution of cocaine or an injection of four grains to the ounce of the aqueous extract of opium.

Dr. Jacobi, of the College of Physicians and Surgeons, states that quite a number of tumors, both benign and malignant, have been found in children. He has himself observed a sarcoma in the kidney of a fœtus 8 months old, and has seen carcinoma of the liver and kidney in the fœtus developed to such an extent as to offer a serious obstacle to the birth of the child. In 1885 he collected all the cases of sarcomata of the kidney in infants, 43 in all at that time, although ten or twelve at least have been reported since. Sarcomata, he says, occurs with greater frequency in the case of young children than carcinomata.

Dr. Sims recently presented, at the New York Polyclinic, a patient that illustrated the influence that an indomitable will sometimes exercises over disease. She had a temperature of 103 1-2 F., and was suffering from the effects of a retained placenta, having had a miscarriage a few weeks previously. She was taken to the hospital and a stream of warm carbolized water was thrown into the uterus, through rubber tubes uninterruptedly for the first 24 hours. The temperature fell to normal as long as the stream was continued, but if interrupted for any time, it rose again to 103 1-2 deg. F. At the end of 48 hours the irrigation was suspended, as the temperature did not again rise, and the uterus was then washed out two or three times a day afterwards. In about ten ten days she left the hospital entirely cured.

The following cough mixture is a favorite one in use at Bellevue Hospital:

℞ Acidi hydrocyani dil., m x x ij.
Morph. sulph. (u. s. sol. gr. j—℥j.)
Syr. pruni virg. ℥j.
M. S. A teaspoonful three times a day. —J. P. R.

